# Patients and family members´ perceptions of interprofessional teamwork in palliative care: A qualitative descriptive study

**DOI:** 10.1111/jocn.16192

**Published:** 2022-01-09

**Authors:** Pauliina Kesonen, Leena Salminen, Elina Haavisto

**Affiliations:** ^1^ Department of Nursing Science University of Turku Turku Finland; ^2^ Department of Nursing Science University of Turku Turku University Hospital Turku Finland; ^3^ Department of Nursing Science University of Turku Satakunta Hospital District Finland

**Keywords:** family members, interprofessional care, palliative care, patient, qualitative research, teamwork

## Abstract

**Aims:**

To describe patients and family members’ perceptions of interprofessional teamwork in specialised palliative care.

**Background:**

Interprofessional teamwork is essential when delivering high‐quality palliative care. Little attention has been paid to patients and family members’ perceptions.

**Design:**

A qualitative descriptive design.

**Methods:**

Semi‐structured individual interviews were conducted with 20 palliative patients and family members (n = 19) in four palliative wards, which were collected from May 2019 to November 2019. Data were analysed using inductive content analysis. COREQ guidelines were followed.

**Results:**

Patients’ perceptions of interprofessional teamwork were described as the nature of interprofessional teamwork, a sense of community and patient participation. Family members’ perceptions of interprofessional teamwork were described as the nature of interprofessional teamwork, the diverse expertise and the sense of community. Patients and family members’ perceptions of interprofessional teamwork were nearly identical and were based on observed social situations or their assumptions. They trust that professionals are working interprofessionally, even if the teamwork cannot be observed. In palliative care, the nature of interprofessional care changes together with patients’ condition and family members progressively need more professional support.

**Conclusions:**

Conducting interprofessional care more openly could benefit the availability of different professionals’ competence to patients and family members. In palliative care, the nature of interprofessional teamwork changes together with the patients’ health condition. More information is needed about what constitutes an interprofessional framework and the required interprofessional competencies in palliative care.

Relevance to clinical practice.

The findings show the importance of considering the patient's health status when interprofessional care is planned. However, professionals should recognise that a patient's weakening condition changes the focus more to the needs of the family members. It is acknowledged that IP teamwork requires time, but in PC settings, spending time on collaborative practices is not always possible.

**What does this paper contribute to the wider global clinical community?**

- Patients and family members’ perceptions are needed for interprofessional teamwork when the goal is to provide high quality and individual patient‐ and family‐centred PC.
- PC, where the care relationship has a specific ending and family members increasingly need support, the IP teamwork has special characteristics compared with other fields of care.
- Interprofessional education is needed to receive all the potential of the team to provide holistic PC without causing discomfort to the patient and family members.

## 1 INTRODUCTION

Annually, over 56.8 million people all over the world are in need of palliative care (PC). This is because of the ageing population and complex health issues. (World Palliative Care Alliance World Health Organization (WPCA & WHO) 2020) PC can be delivered in different clinical settings from home to hospitals. Specialised PC services are needed when patients’ have complex care needs, and the expertise of different professionals is essential to deliver holistic care (Gamondi et al., 2013). In PC, the aim is to ensure the quality of life of patients whose death is imminent and their families (WPCA & WHO, 2020). An IP approach should combine different professionals, patients and their families together to deliver quality care (WHO, 2010).

With the collaboration of different professionals, it is possible strengthen healthcare delivery and also achieve better health outcomes (World Health Organization (WHO) 2010). At the core, interprofessional (IP) teamwork is the activity of the professionals. However, the goal of the team is to provide quality PC for the patient and their families. An IP team can consist of several different professionals, and being a competent team member means acquiring competencies that are both discipline‐specific and relevant to all those joining the IP team. Elements such as quality of life, the relationship between patients and professionals and a multiprofessional approach are described as essential in PC settings. In addition, ethical aspects such as the patient's situation as regard life and death, autonomy and dignity are always present. To achieve better care outcomes, an extensive IP team is probably not necessary in PC settings. It is more important that the team shares the same philosophy and has a joint understanding of the care goals. In specialised PC, more qualified professionals work exclusively with complex issues relating to PC. (Gamondi et al., 2013.)

It is suggested that patients and families are involved in both the IP teamwork (WHO, 2010) and the PC (WPCA & WHO, 2020). Despite the recommendations, there have been very few studies that combine IP teamwork and patient and families’ perceptions. The role of the patient and family in PC has been discussed but as regard teamwork, the patients and their family's position are unclear, and their roles are poorly documented (McDonald & McCallin, 2010). In this study, IP care is defined as a process where the roles of different professionals in the care process are specialised, but they are expected to interact (Klarare et al., 2013). The focus of this study is on specialised PC wards, where the main activity is to produce quality end‐of‐life care for the patients with more complex care needs by utilising the expertise of multiple professionals (Gamondi et al., 2013; Hui et al., 2018).

## 2 BACKGROUND

IP care has been highlighted when delivering PC (European Association for Palliative Care (EAPC) 2020; Hui et al., 2018; McDonald & McCallin, 2010) and professionals from different disciplines are required to work together for the good of patients receiving PC and their families (Connolly et al., 2016; McDonald & McCallin, 2010; WHO, 2010). Because of the special nature of PC, different professionals’ participation in the care goal discussions are essential and all professionals play an important role in patient care (You et al., 2015). Although patient‐centred care is designated as one of the key features in IP teamwork (WHO, 2010), it might be challenging, even unrealistic in life‐limiting situations (Sanderson et al., 2017). It is known that it might be challenging for patients and family members to accept bad news or difficult to understand the limitations of available treatments (You et al., 2015). Moreover, the possibility for patients to participate in care meetings might be limited because of their current health status. In some cases, like communication, the role of family members might be even greater than the patients. (Sanderson et al., 2017.) Whether or not a patient and the family want to be involved, care should be conducted interprofessionally and holistically.

To deliver PC effectively in IP teams, professionals should be competent in their own profession but also interprofessionally (Witt Sherman et al., 2017). IP competencies are needed when professionals are working with other professionals, patients, families and organisations (Canadian Interprofessional Health Collaborative (CIHC) 2010; Interprofessional Education Collaborative (IPEC) 2016). IP teamwork consists of certain types of elements such as the clarification of the professional members’ role in the team, patient‐ and family‐centred care, communication (CIHC, 2010; Hepp et al., 2015; IPEC, 2016; WHO, 2010; Wood et al., 2009) and shared documentation (Wilhelmsson et al., 2012; Wood et al., 2009). Because of the nature of PC as regard the elements mentioned previously certain types of professional attributes are preferred, such as respect, empathy (Ciemins et al., 2015), sensitivity and communication skills (Ciemins et al., 2015). IP teamwork correlates patient–staff communication and patients may experience the execution of communication in a different way compared with the professionals (von Knorring et al., 2020).

Patients and their family members as part of the team and in team meetings have been studied in PC (Sanderson et al., 2017), but to the best of our knowledge, the literature reveals gap concerning how patients and family members experience IP teamworking during PC in hospital settings. Although the goal of PC is to provide quality care for the patient and their families with the help of different professionals working in IP teams (Gamondi et al., 2013), little is known about how patients and family members experience IP teamworking in PC; therefore, this study aims to describe patients and family members’ perceptions of IP teamwork in a specialised PC ward. The ultimate goal was to add knowledge of IP teamwork in specialised PC delivery from the point of view of patients and their family members in order to promote care delivery and patient safety and also to encourage the systematic involvement of patients and their family members in IP teamworking. This study is a part of a larger study considering evidence‐based practices and the competence of professionals in palliative and end‐of‐life care; the aim of the larger study is to improve quality of care and patient safety.

## 3 METHODS

### 3.1 Aim

Aim of this study was to describe patients and family members’ perceptions of IP teamwork in a specialised PC ward.

### 3.2 Design

A descriptive qualitative design with semi‐structured individual interviews was used to describe the interprofessional teamwork comprehensively from patients’ and family members’ perspectives. Data were inductively analysed at the surface and manifest level on the experiences of patients and families in their own words. (Sandelowski, 2010.) The Consolidated Criteria for Reporting Qualitative Research (COREQ checklist) was followed when preparing the manuscript (Tong et al., 2007) (Supplementary File 1).

### 3.3 Participants

A purposive sampling strategy was adopted (Elo et al., 2014) to recruit both patients and family members. Four different specialised PC wards in four different hospitals in the south and southwestern parts of Finland were chosen, because approximately 39% (2.2 million) of all Finnish people live in this area (Official Statistics of Finland (OFS) 2021). Specialised PC units are responsible of taking care of patients who are incurably ill, and their end‐of‐life care needs are complex and intense requiring the specialised competencies of professionals (Gamondi et al., 2013).

The suitability of the patient was assessed individually by the PC ward staff. The inclusion criteria for patients were as follows: 18 years old or older, Finnish‐speaking, inpatient, patient has received palliative hospital care for at least one week, are incurably ill, receiving only symptomatic not delaying treatments and good fundamental care, and is capable of willingly sharing her/his perceptions. The patient and the family members participated separately, consequently their family relationship was not determined. The patient named the family member who could be asked to join the study. Participation of the patient was not a prerequisite for the participation of the family member and vice versa.

### 3.4 Data collection

Before starting the data collection, researchers met the ward staff and provided them with information about the whole research project in general and recruitment process (e.g. the criteria for suitable participants). The ward staff gave oral and written information about the study to the possible participants and asked them if they would take part in the study; those who participated gave their written informed consent and returned it in a sealed envelope. The contact person, who was a registered nurse on the ward, informed the interviewer when it was suitable to conduct the interviews.

Data were collected between May and November 2019 by two interviewers interviewing participants individually. All participants were interviewed face‐to‐face except for one relative who was interviewed over the telephone. Interviews were undertaken in clinical settings. A calm place on the ward was requested in which to conduct the interviews to minimise distractions and to obey the duty of confidentiality; the place was chosen by the interviewee or the ward staff. Only the interviewee and interviewer were present.

The semi‐structured interviews were based on previous framework describing the goal of the IP team and competencies of professionals working in PC to provide quality care for the patient and their families (Gamondi et al., 2013). All interviews were digitally audio‐recorded with the consent of the interviewee and were transcribed verbatim. The participants were asked to describe their perceptions of the following: (1) which professionals have participated in their care in this current ward, (2) have these professionals worked as a team/collaboratively in care and (3) how can the teamwork be observed and described. Supportive questions were asked, and concrete examples were requested along with the initial questions. The interview guide was pilot tested, and these data were also included in the final data as there were no need for changes.

The interviews were conducted between May and November 2019. Patients’ interviews lasted approximately 24 minutes (ranging from 9 minutes to 50 minutes) and family members’ interviews lasted approximately 33 minutes (ranging from 13 minutes to 1 hour 13 minutes). Out of respect for the patient's health condition and family members’ well‐being, the interviews were highly focused and relatively short in duration. In three cases, the interviews were interrupted due to treatment being needed for the patient and the patient's health condition. In some cases, the patient's health condition challenged the data collection (e.g. their mouth was so dry, it was hard to understand their speech or incoherence caused by a brain tumour). The participants’ background information (age, gender, illness, the length of the illness and the length of the treatment) was requested orally at the beginning of the interview.

### 3.5 Data analysis

The interviews were analysed using inductive content analysis because it is applicable when studying perceptions (Graneheim et al., 2017). The analysed data only consisted of the interview material and the data from the patients and the family members’ interviews were analysed separately based on the aim of the study (Kyngäs et al., 2020). The data analysis process was conducted individually by one researcher (PK) and checked by two other independent researchers (EH and LS); it was also discussed with the research group. The analysis process was conducted through the following phases (Table 1): At first, the interview material was read through several times to become familiar with the content. The data were examined to find units for analysis, and then, relevant expressions corresponding to the aim of the study were identified from the transcripts. The selected sentences were simplified by removing unnecessary words. Expressions were coded and compared to identify similarities and differences and based on this comparison, grouped into subcategories by inductive category forming. The classification was continued by summarising the subcategories into the categories (Lindgren et al., 2020.). Data saturation was achieved with 20 patients and 19 family members (Elo et al., 2014).

**TABLE 1:** An example of the analysis process

Original expression	Simplification	Subcategory	Category
‘It is really hard for comment because I have not needed the help of any other professional’.	It is hard to comment if the help of certain professionals has not been needed.	Patient‐centred palliative care	Patient involvement
‘Physiotherapist came to see me, but at this point I don´t need anyone to come here’.	Patient experiences that there is no need for meeting physiotherapist.		
‘Professionals have asked if I want to, but it is hard to stay awake and keep my eyes open. It is important to control the pain first, so I can think’.	Patient is too tired to participate on IP care planning.	Health status‐dependent teamwork	
	Pain effects negatively on patients thinking.		
‘The library personnel and the singer came on the same day, and there were those volunteers too. There were too many people, so I said nicely to the volunteer, that “thank you kindly, but my things are arranged well this moment’.	Having too many contacts have its effect on patients’ ability to participate.		
‘Actually, this whole process has proceeded so quickly that just a few professionals have been participating my care’.	The disease has proceeded so quickly that only a few professionals have participated in the care.		

## 4 ETHICAL CONSIDERATIONS

This study was conducted according to the Declaration of Helsinki (World Medical Association (WMA) 2013). Ethical approval was requested to undertake the study and was received from the Ethics committee for Human Sciences at the University of Turku (15/2019). In addition, permissions were requested and obtained from the organisations from which the data were collected. All participants received oral and written information concerning the ongoing study. Participation was voluntary, anonymity was guaranteed during the whole research process and participants provided informed written consent for their participation in the study. The participants were informed of their right to withdraw their participation at any time, and confidentiality was observed at all times during the study. A written information letter including the researchers’ contact information was provided, and the participants had an opportunity to ask questions when receiving the oral information and also during the interviews. The participants were informed that participating in the study would have no effect on the care they were receiving. Extra attention was paid to the vulnerability of the participants. The terms used were chosen to be as neutral as possible, and the interviewers endeavoured to keep the length of the interviews short. The well‐being of the participants was monitored during the interviews, and interview was suspended with a low threshold.

## 5 RESULTS

### 5.1 Characteristics of participants

A total of 20 patients and 19 family members from four different specialised PC wards in Finland participated in this study (Table 2). Nine of the family members were spouses of the patient, five were children, two were friends, and in one case, each a sister, a mother and a father. Seventeen of the participating patients were suffering from an incurable cancer (different types), and the diagnosis of three patients was unknown or not mentioned. The relatives of the family members who participated in the interviews were all suffering from some form of cancer, except one patient who had ALS.

**TABLE 2:** Characteristics of the participants

Characteristics	Patients	Family members
Total	20	19
Females	13	11
Males	7	8
Age (in years)		
Mean	75.3	61.6
Range	62–94	41–80
Time from diagnosis		
Mean	2.8 years	1.4 years
Range	two months–17 years	2 months–8 years
Treatment period at the current unit		
Mean	2.8 weeks	5.7 weeks
Range	1–6 weeks	1.5 weeks–6 months

### 5.2 Patients’ perceptions of interprofessional teamwork in specialised palliative care

Patients’ perceptions of interprofessional teamwork in specialised palliative care were described as follows: *Nature of interprofessional teamwork*, *Sense of community and Patient involvement* (Table 3).

**TABLE 3:** Patients’ perception of interprofessional teamwork in specialised palliative care

Subcategory	Category
Visible interprofessional teamwork	Nature of interprofessional teamwork
Invisible interprofessional teamwork	
Mutual communication	Sense of community
Team spirit	
Patient‐centred palliative care	Patient involvement
Health status‐dependent teamwork	

The *Nature of interprofessional teamwork* consists of visible interprofessional teamwork and invisible interprofessional teamwork. Participants identified different social and healthcare professionals participating in their care. The majority of the patients and family members identified nurses and medical professionals, and approximately half of the participants named physiotherapists. Additional professionals identified were priests, social workers, occupational therapists, nutritional therapists, ward domestics, kitchen personnel, volunteers and crisis centre workers. Many patients had the impression that IP teamwork was working well or very well on the ward. Teamwork among medical and nursing professionals was most commonly described. Patients described good teamwork as being demonstrated by nothing strange happening on the ward or there is no need for changes; these images give the impression that the teamwork is functional. According to the patients, visible IP teamwork means both working as a group and working as an individual.

*‘You are doing that and I´m doing this and this we are doing together, and all professionals are participating’*.

Patients described invisible interprofessional teamwork in two ways; either the teamwork is not noticeable or the participating professionals cannot be identified. Two patients described that a lack of problems gave them the feeling that there are no problems as regard the IP teamwork. Identifying different professionals caused some problems. For patients, it is hard to remember the names of the professionals and they are not aware which professionals are participating in their care, because their similar clothing makes it harder to identify one professional from another.

*‘Actually, I have never thought which professionals they are, because I can´t even remember their names’*.

The second category *Sense of community* consists of two subcategories: mutual communication and team spirit. When patients can see that professionals are communicating directly with each other or they have a feeling that the professionals have an instant connection to one another, it is a sign of collaborative working. When patients see nurses and physiotherapists or nurses and doctors communicating, it gives an impression of IP teamwork. Two patients identified electronical databases as one kind of opportunity to collaborate. Patients described communication among different professionals also as conveying messages between professionals:

*‘I do trust, or at this point I have not noticed any problems. Whatever has been asked has been conveyed to the doctor. The doctor has received my requests’*.

Team spirit occurred in two ways: the atmosphere in the ward and supporting one another at work. Patients felt that a good general atmosphere is experienced when there is solidarity, happiness, laughing and a lack of arguing among professionals. The feeling that professionals are working well together and getting along with each other gives the impression of a good team spirit. One patient described that solidarity is a prerequisite for teamwork and if there is the opposite, a sense of a hierarchy on the ward, there is a lack of team spirit. Professionals supporting one another at work means being on the same side and taking care of one another gives an impression of a good team spirit. One patient described IP teamwork as help given across professional boundaries. Patients were able to ask help from ward domestics if the task was a common care‐related task.

*‘Yes, this is completely clear. Care and teamwork cannot work if there is no team spirit’*.

*‘In my opinion it is clearly working. Like they are taking care of each other. It clearly can be seen, and it feels that the personnel are not arguing with each other’*.

The third category *Patient involvement* consists of two subcategories: patient‐centred palliative care and health status‐dependent participation in teamwork. When different professionals have interprofessionally helped the patient and the task division is clear, patients described the care as having more quality. Several patients found it was easier to list the professionals whose help they did not need. For example, if they have been offered a visit from a social worker or physiotherapist, but the patient did not see the need. In several cases, nurse–doctor collaboration was felt to be sufficient, and sometimes meeting physiotherapist was also useful. Generally, it was hard for the patients to comment when they had not needed or wanted the help of several different professionals.

*‘It is really hard for comment because I have not needed the help of any other professional. Of course, there are those nurse aide´s or how you currently call them’?*

As regard health status‐dependent teamwork, the patients often described that they were too tired, sick or in severe pain to participate in teamwork. Health‐status dependent teamwork was present in patients’ perceptions. End‐of‐life care gives special input to IP teamworking when the diagnosis has been given with a very short notice or the prognosis is very bad or the disease itself has rapidly deteriorated. A few patients described the IP in PC as demanding, because of the many contact people and tight schedules, which often meant that all the contacts were on the same day. In these cases, patient felt that some of the professionals needed to be excluded.

*‘Professionals have asked if I want to, but it is hard to stay awake and keep my eyes open. It is important to control the pain first, so I can think’*.

*‘The library personnel and the singer came on the same day, and there were those volunteers too. There were too many people, so I said nicely to the volunteer, that “thank you kindly, but my things are arranged well this moment’*.

### 5.3 Family members’ perceptions of interprofessional teamwork in specialised palliative care

Family members’ perceptions of interprofessional teamwork in specialised PC can be described as *Nature of interprofessional teamwork in specialised palliative care*, *Diverse expertise* and *Sense of community* (Table 4).

**TABLE 4:** Family members’ perception of interprofessional teamwork in specialised palliative care

Subcategory	Category
Visible interprofessional teamwork	Nature of interprofessional teamwork
Invisible interprofessional teamwork	
Complementary expertise	Diverse expertise
Clarity of roles	
Mutual communication	Sense of community
Team spirit	
Possibility to participate in teamwork	

The first category describes the *nature of interprofessional teamwork* in specialised PC consisting of two subcategories: visible interprofessional teamwork and invisible interprofessional teamwork. Family members described professionals as working as a team and every professional making an effort to get things done. Family members saw doctors and nurses at the centre of teamwork, but they also described teamwork among just one group of professionals, such as the nurses, as helping each other in care situations. Invisible teamwork can occur in two ways; when the teamwork cannot be noticed, or there is a lack of IP teamwork. A few family members could not comment on the teamwork question because the teamwork in the care unit could not be seen. Teamwork was also hard to describe because they did not know which professionals were participating and which one was not involved. Two family members described that there was no teamwork between different professionals. They said it appeared that professionals were only doing the work appointed to them, but nothing else.

‘It is hard to comment because nurses and doctors are my contact. It is hard for me to say, which other professionals there are in the background’

‘There is no kind of teamwork. Everyone is doing the job appointed to them’.

*Diverse expertise* consists of two subcategories: complementary expertise and clarity of roles. Family members had the impression that there were many different professionals working in specialised PC and all were complementing each other's knowledge and helping the patients and their family. Family members described the professionals as being competent and working for the good of the patient; however, it was hard to specify what kind of knowledge professionals need to work as an IP team. Because of the nature of PC, family members respected the different qualities of every professional participating in the IP team, particularly those such as empathy and their understanding of human nature.

*‘Quite an army is taking care of this circus’*.

*‘These education and profession things are really unclear to me. I don´t know is it important for me to know these different titles. But I think there are more than one profession participating’*.

The clarity of roles in IP teams appeared in the way the actual work was organised in practice. It is important that the right tasks are transmitted to the correct professional and every professional should take care of their own tasks. Some family members felt that the occupation of the IP team members was unclear. It is hard to specify whether an individual is a registered nurse or a practical nurse. Sometimes, the profession can be inferred from the tasks the professionals are performing or from the name tag on their uniform.

*‘Of course, it is written on the nametag, whether they are nurses or practical nurses. But I haven´t been staring at those tags. I think that in work like this, of course the education is important. Thank God in Finland education is required’*.

*A sense of community* consisted of three subcategories: mutual communication, team spirit and the possibility to participate in teamwork. Family members described mutual communication as an action where different professionals were seen talking to each other on the ward. During communication, information regarding a patient's care is both exchanged and transmitted. Family members felt that professionals need to know what is happening with the patient and, for example nurses were made aware of the content of conversations between family members and doctors. Two family members described electronical databases as being one way of exchanging information between professionals. Additionally, the situations where the communication was not successful were described. Cases where the professionals did not discuss with each other or told family members that communication with other professionals was not part of their job.

*‘The doctor said that it is not their job. So, I can clearly say, that professionals are not communicating because it is not their business’*.

Family members described the team spirit among IP team. They felt that the atmosphere was warm, and everyone was getting along well. When conflicts or arguments were not noticeable, it gave an impression of a good team spirit. In addition, they felt that the number of years the professional had worked together helped ‘the team feeling’, as one family member described:

*‘Probably they have worked together for many years. The encounter was warm. And even if the patient is present, the atmosphere was really warm’*.

The patients and family members’ possibilities to participate in the teamwork were also the way that they sensed interest and enthusiasm from the professionals on the ward. They compared their previous experiences and felt that in the current palliative ward, the professionals, in general, were participating more actively. Considering their own participation in IP teamwork, family members had both experiences; they have had the possibility and not had the possibility to communicate with the professionals. Family members felt that the nature of IP teamwork changes when the patients’ health status changes. At first, patients are more interested in participating in teamwork, but when the disease progresses and the patient's well‐being is deteriorating, family members are the people who need more support from the professionals.

*‘When you recognise that the condition of the relative is getting worse and we are not able to manage with the situation anymore or don´t know what should do. Then some kind of contact at least is needed, how to start the evaluation process to find out if more support is required’*.

## 6 DISCUSSION

The aim of this study was to describe patients and family members’ perceptions of IP teamwork in specialised PC. Overall, the perceptions of both groups had similar elements but also varied to some extent. Both described the nature of IP teamwork in similar ways, whether it is visible or invisible and somewhere in the background. They also described more feelings or assumptions rather than concrete examples of the ways in which the teamwork was conducted. A few family members conveyed the perception that there is clear lack of IP teamwork. The results do not indicate whether the IP teamwork is invisible or totally lacking, yet the finding of the teamwork visibility should be more discussed in PC where IP teamwork is highly recommended for the good of the patient and their families (Gamondi et al., 2013; McDonald & McCallin, 2010; WHO, 2010).

Patients and family members’ perceptions had similar elements as regard the sense of community. Moreover, team spirit was recognised in both interviews. Patients and family members appreciate a proper exchange of information between professionals. When delivering bad news (You et al., 2015) transparent IP teamwork might be one way to show that professionals are there until the end and working diligently for the best for the patient and their family members. Communication has been described as one of the key skills in IP teamwork (Hepp et al., 2015; Wilhelmsson et al., 2012; Wood et al., 2009) yet it is known that patients can experience communication differently when compared to the experience of professionals (von Knorring et al., 2020). Studying this aspect by comparing the perceptions of patients, family members and professionals would give valuable information about IP teamwork. Although the perceptions were nearly alike, only family members connected patient and family member participation to the IP teamwork as being important, and to be recommended (Gamondi et al., 2013; WHO, 2010). Another difference between patients’ perceptions and family members was that the family members focused more on the clarity of the professional's role (CIHC, 2010; Hepp et al., 2015; IPEC, 2016; Wood et al., 2009), probably because in many cases, family members have been actively participating in care and taking care of their close one for a long time. Family members also focused on how knowledge in the IP team was organised and if the correct professionals were working with right care‐related tasks.

The most remarkable difference between the perceptions were that the patients focused on the quality of the care provided (Gamondi et al., 2013; Hepp et al., 2015; Wood et al., 2009), while family members were more focusing on the diversity and complementary expertise of the professionals. The result is understandable, as patients are the recipients of the care given and the family members are the bystanders, participating when possible. From the patients’ perceptions, it can be seen that the patient's health status has an effect on the IP teamwork (Sanderson et al., 2017). When the patient is incurably ill and a prognosis for the future health status cannot be given, naturally it influences how the IP teamwork was seen or experienced. For family members, there might be difficulties to let go of active and rehabilitative care and focus more on the quality of symptomatic care. In general, the IP care frameworks highlight active participation from patients and family members (CIHC, 2010; IPEC, 2016). The IP approach is also seen as positive and self‐evident, but in PC, this may not be the case. This study does not give answers as regard the place of patients or families in a PC team, or whether they should be willing to participate in a team (McDonald & McCallin, 2010). The health condition and vulnerability of the patients should also be noted when planning and implementing IP care. Extra attention is needed to recognise the limit where the care is considered to be of help or has become too demanding for a severely ill patient. While conducting the interviews, a few agreed interviews were cancelled because of the changes in the patients’ condition or because they had died. In PC, the presence of death challenges the IP teamwork, because it creates more pressure on an already time demanding process.

In PC, the relationship between patients and professionals has relatively different ending compared with other care settings, and IP teamwork should be delivered by noticing the care context (WHO, 2010). When developing care delivery competencies need to be defined. In the absence of an existing framework, it was essential to describe the patients and their family members perspective of IP teamwork as the goal in PC is to meet the patients and their family members as individuals until the end‐of‐life (Connolly et al., 2016). In comparison with previous IP frameworks (CIHC, 2010; IPEC, 2016; WHO, 2010) and recommendations in PC (EAPC, 2020; Gamondi et al., 2013; WHO, 2010), this study gathers elements from both sides; how to work interprofessionally to promote the quality of life of patient with life limiting health conditions and their family members. However, the results are unilateral and therefore need confirmation from different perspectives. It is understandable that the participants in this study were not able to describe IP teamwork in the way that it is presented in the literature (e.g. describing the team philosophy or joint understanding of the care goals); nevertheless, the results give an impression of the elements valued in IP during PC.

Overall, describing IP teamwork was hard for both patients and family members. In some cases, it was felt that the participants were describing how different professionals were collaborating with a patient or/and a family member, not how different professionals were collaborating as a team in which the patient and family members were participants. In general, patients and family members trust that professionals are working interprofessionally even if the teamwork is not observable. Instant communication between professionals and a warm and happy atmosphere on the ward give the impression of good IP teamwork. However, it is known that patients can experience communication differently when compared to the experience of professionals (von Knorring et al., 2020). Although different professionals are participating in the care and an IP approach is highly recommended, the nurse–doctor collaboration was still the one most described.

## 7 STRENGTHS AND LIMITATIONS

The strengths and limitations of this study were demonstrated through credibility, transferability, dependability and confirmability according to the Guba and Lincoln criteria (Schwandt et al., 2007). Conducting interviews with two interviewers had an impact on the data collection. Minimising the possibility of differences, several orientation sessions were held to familiarise the interviewers with questions. Nevertheless, the choice to use two interviewers enabled the data collection in different PC units in different regions. Individual preconceptions or the previous experiences of the researchers might have created bias during the research process. In addition to the ongoing research, the interviewers did not have any other association with the participating PC wards, suggesting that they did not have any influence on the descriptions received. Researchers have sought to minimise the impact of own assumptions and experiences in the data analysis process. The analysis process was checked by two independent researchers, then the final output was discussed and verified by the research group to increase the trustworthiness of the analysis (Willis et al., 2016). Nonetheless, qualitative descriptive study is interpretative to some extent (Sandelowski, 2010). The credibility of the research was increased by using direct quotations when reporting the study findings.

The transferability of the study findings was enabled by describing participants´ demographic information. Geographically, the interviews were held in the southern parts of Finland where the population density is higher compared to other parts of the country. The study context and inclusion criteria for participation were described as precisely as possible to enable the transferability of the results. Furthermore, some of the descriptions began to recur which were considered a sign of a saturation. (Elo et al., 2014.) All these factors allow readers to assess the possibility to transfer the results to another context after careful consideration.

Dependability was confirmed by giving detailed information about the aim of the study, describing the participant recruitment and data collection process. Moreover, the data analysis process was described phase by phase and supported with tables to provide transparency. Hence, during the recruitment phase, providing only the main goals of the ongoing study minimised any possible changes in professionals’ behaviour during data collection.

Confirmability was increased by asking additional questions or for clarification during interview if necessary and also by using the expertise of the research group in the data analysis process. Repeat interviews were not carried out nor were transcripts returned for comments/feedback, because of weakening condition of the patients. (Schwandt et al., 2007.) In addition, few agreed interviews, both with patients and family members, were cancelled because of the changes in the patients’ health status or because they were died.

## 8 CONCLUSION

The perceptions of patients and family members had similar elements but also varied to some extent. The results indicate that IP teamwork could be delivered in a more visible manner and both the patients’ and family members’ participation could be enhanced to meet the PC recommendations. It seems that in specialised PC, where the patients care needs are more complex and intense, observing patients’ health status more closely is important. The nature of IP teamwork and the family members’ role in the team changes as the patients’ health status changes. Understandably, balancing between patients’ weakening condition and the family members’ emerging awareness of the coming loss is hard for professionals working in PC settings. As regard differences, patients tended to focus more on the quality of care while family members drew attention to the clarity of the role of the professionals. When planning PC, the care should be tailored to the patient, so that the IP care received is individual to the end. Future research should focus more on interventions in order to establish a suitable IP framework which considers all the special features of PC. Additionally, measuring objectively the level of IP competence of professionals working in specialised PC would be beneficial to clearly defining the current state of IP capability.

## 9 RELEVANCE TO CLINICAL PRACTICE

The results of this study are important for clinical practice as they can help IP teamwork to deliver more holistic and patient‐ and family‐centred care in PC. IP care should be delivered transparently and, patients and family members should be involved more systematically while also noticing the care context and patient's current condition. In PC settings, patients’ health status should be considered when planning IP care. Moreover, clear and seamless teamwork structures are essential. It is important that management facilitates a collaborative atmosphere in organisations working in specialised PC. Health and social care professionals need appropriate continuing education with regard to IP teamwork when working in PC. Improving the IP competence of professionals working in PC would benefit the care delivery in end‐of‐life related situations. However, the care goals differ to some extent when compared to other fields of care.

## CONFLICT OF INTEREST STATEMENT

The authors declare that they have no conflict of interests.

## Supporting information

Supplementary Material
